# Human Traumatic Brain Injury Induces Autoantibody Response against Glial Fibrillary Acidic Protein and Its Breakdown Products

**DOI:** 10.1371/journal.pone.0092698

**Published:** 2014-03-25

**Authors:** Zhiqun Zhang, J. Susie Zoltewicz, Stefania Mondello, Kimberly J. Newsom, Zhihui Yang, Boxuan Yang, Firas Kobeissy, Joy Guingab, Olena Glushakova, Steven Robicsek, Shelley Heaton, Andras Buki, Julia Hannay, Mark S. Gold, Richard Rubenstein, Xi-chun May Lu, Jitendra R. Dave, Kara Schmid, Frank Tortella, Claudia S. Robertson, Kevin K. W. Wang

**Affiliations:** 1 Department of Psychiatry, Center for Neuroproteomics and Biomarker Research, University of Florida, Gainesville, Florida, United States of America; 2 Banyan Biomarkers Inc., Alachua, Florida, United States of America; 3 University of Messina, Messina, Italy; 4 Department of Anesthesiology, University of Florida, Gainesville, Florida, United States of America; 5 Clinical and Health Psychology, University of Florida, Gainesville, Florida, United States of America; 6 Department of Neurosurgery, University of Pécs and Clinical Neuroscience Image Center of Hungarian Academy of Sciences (HAS) Pécs, Hungary; 7 Department of Psychology, University of Houston, Houston, Texas, United States of America; 8 Laboratory of Neurodegenerative Disease and CNS Biomarkers, Departments of Neurology and Physiology/Pharmacology, SUNY Downstate Medical Center, Brooklyn, New York, United States of America; 9 Center for Military Psychiatry and Neuroscience, Walter Reed Army Institute of Research, Silver Spring, Maryland, United States of America; 10 Department of Neurosurgery, Baylor College of Medicine, Houston, Texas, United States of America; Weizmann Institute of Science, Israel

## Abstract

The role of systemic autoimmunity in human traumatic brain injury (TBI) and other forms of brain injuries is recognized but not well understood. In this study, a systematic investigation was performed to identify serum autoantibody responses to brain-specific proteins after TBI in humans. TBI autoantibodies showed predominant immunoreactivity against a cluster of bands from 38–50 kDa on human brain immunoblots, which were identified as GFAP and GFAP breakdown products. GFAP autoantibody levels increased by 7 days after injury, and were of the IgG subtype predominantly. Results from in vitro tests and rat TBI experiments also indicated that calpain was responsible for removing the amino and carboxyl termini of GFAP to yield a 38 kDa fragment. Additionally, TBI autoantibody staining co-localized with GFAP in injured rat brain and in primary rat astrocytes. These results suggest that GFAP breakdown products persist within degenerating astrocytes in the brain. Anti-GFAP autoantibody also can enter living astroglia cells in culture and its presence appears to compromise glial cell health. TBI patients showed an average 3.77 fold increase in anti-GFAP autoantibody levels from early (0–1 days) to late (7–10 days) times post injury. Changes in autoantibody levels were negatively correlated with outcome as measured by GOS-E score at 6 months, suggesting that TBI patients with greater anti-GFAP immune-responses had worse outcomes. Due to the long lasting nature of IgG, a test to detect anti-GFAP autoantibodies is likely to prolong the temporal window for assessment of brain damage in human patients.

## Introduction

Traumatic brain injury (TBI) is a leading cause of death and disability worldwide, with approximately 2 million reported TBI events in the US annually. The pathogenesis of TBI involves two components: the initial mechanical injury, and subsequent secondary cell death that expands the core lesion [1], [2]. During acute neuronal necrosis, calpains are hyper-activated, while caspases are activated in apoptosis [3],[4]. Animal model studies and clinical data both indicate that blood-brain barrier (BBB) breakdown frequently follows head trauma [1],[2],[5]–[7]. Cell death within the first day following TBI promotes release of brain proteins and their breakdown products (i.e., putative biomarkers) from injured cells into biofluids such as cerebrospinal fluid (CSF) and blood [2]–[4],[8]. Identified biomarkers observed in human biofluids post TBI include neuron specific enolase (NSE), glia calcium-binding protein S100B, glial fibrillary acidic protein (GFAP), myelin basic protein (MBP), ubiquitin carboxyl hydrolase-like 1 (UCH-L1), neurofilament proteins, and αII-spectrin breakdown products (SBDPs) [9]–[13]. Quantitative detection of these biomarkers in biofluids would support a relatively simple and straightforward means of detecting brain injury. Because TBI diagnosis currently relies primarily on MRI and/or CT scans and neurological assessments, blood-based biomarker tests would represent a valuable new clinical tool [14].

After TBI and rupture of the BBB, brain proteins (potential biomarkers) released from damaged brain cells enter the bloodstream where they may trigger an immune response. This study reports the results of a primary screen to determine which brain biomarkers become targets of the immune system after TBI. Autoimmunity involves the development of antibodies against self-antigens, or autoantibodies. Depending on subtype, antibodies can be maintained within the bloodstream for years. Multiple sclerosis (MS) is an example of an autoimmune disease that involves a central nervous system (CNS) antigen. Patients with MS develop circulating autoantibodies against MBP [15]. Reports have documented brain-directed autoimmunity in neurological and neurodegenerative diseases such as Alzheimer’s disease, stroke, epilepsy, and paraneoplastic syndromes [16]–[20]. Additional studies have reported autoimmune responses in spinal cord injury [21]–[24].

In human TBI, however, autoimmunity has only been examined in a limited way and focused on autoantibodies against preselected antigens such as MBP, S100B, and glutamate receptors [15],[25]–[28]. Tanriverdi et al. also identified the presence of anti-pituitary antibodies in patient serum 3 years after head trauma [29],[30]. Mostly recently, Marchi et al. showed that college American football players may experience repeated BBB-disruption and serum surges both S100b and subacute auto-S100B antibodies. They further identified a correlation of serum S100B, auto-antibodies and white matter disruption [31]. In the present study, we employed a global, systematic neuroproteomics approach to discover the predominant brain autoantigens associated with human TBI. The study set included 53 patients with severe TBI, and age-matched healthy controls. Serum samples from each individual collected on days 0–10 post TBI were screened against human brain lysate by western blotting to visualize autoantigens. Then tandem mass spectrometry was used to identify the autoantigens. Remarkably, TBI patients developed autoantibodies that were directed primarily against GFAP and its breakdown products (BDP). GFAP is an intermediate filament protein specifically localized to the cytoskeleton of mature astrocytes, the most abundant cell type in the central nervous system [32]. This report represents a characterization of anti-GFAP and GFAP-BDP autoantibodies post TBI and an initial evaluation of their clinical relevance. We hypothesize that post-translational modified GFAP could breakdown the self-tolerance and preferably serve as the immunodominant autoantigen to trigger autoimmune response following TBI. In addition, anti-GFAP autoantibodies might also provide the basis for a novel, blood-based diagnostic test for human TBI in the subacute phase.

## Materials and Methods

### Human Study and TBI Patient Population

All research involving human participants had protocols approved by individual author’s respective Institutional Review Board (University of Florida Institutional Review Board, Baylor college Institutional Review Board and University of Pecs Institutional Review Board) at each location. The local ethics and hospital management committees also approved the protocols. Informed consent was written. Archived deidentified biosamples (serum) are used for this study. Samples from adult severe TBI patients were analyzed retrospectively. Patient serum samples were from Baylor College of Medicine (Houston, TX; n = 16), University of Florida (Gainesville, FL; n = 10) and University of Pécs (Pécs, Hungary; n = 27). To be considered a TBI patient, the following criteria had to be met: Patients >18 years of age with severe TBI defined as having a sum Glasgow coma score (GCS) of ≤8 on the post-resuscitation admission neurological examination and requiring a ventriculostomy catheter for clinical management. Serial serum samples were collected from enrollment to up to day 10 from 53 severe TBI patients. The number of blood samples per patient ranged from 2–11. For controls, 96 volunteers at least 18 years of age with no history of significant trauma were recruited under informed consent, unless falling under exclusion criteria. Exclusion Criteria: 1) No ventriculostomy needed or could not be placed; 2) Life-threatening systemic injury (AIS = 5) in any organ system other than head or spine; 3) Severe pre-existing chronic disease; 4) History of severe psychiatric illness (e.g., schizophrenia); 5) History of prenatal retardation. One blood sample was collected per control individual. All patient identifiers were kept confidential.

### Collection of Outcome Data (i.e. GOS-E)

Patient GOS-E scores were assigned to dichotomous “favorable” vs “unfavorable” outcome groups according to recommendations and conventions extensively reported in the literature [33],[34]. The “favorable outcome” group was comprised of Upper/Lower Good Recovery and Upper/Lower Moderate Disability, while the “unfavorable outcome” group was comprised of Upper/Lower Severe Disability, Persistent Vegetative State, and Death.

### Animal Work

All animal work have been approved by and conducted according to the policy of the University of Florida Institutional Animal Care and Use Committee (IACUC) or the Walter Reed Army Institute of Research (WRAIR) Institutional Animal Care and Use Committee (IACUC). In addition, all experiments were done in a manner consistent with the NIH Guide for the Care and Use of Laboratory Animals. For the penetrating ballistic-like brain injury (PBBI) model, rats were generated at the Walter Reed Army Institute of Research (WRAIR), and the WRAIR IACUC approved all experimental procedures. PBBI was induced on anesthetized male Sprague-Dawley rats (Charles River) by stereotactically inserting a perforated steel probe through the right frontal cortex of the anesthetized rat and rapidly inflating the probe’s elastic tubing into an elliptical shaped balloon to 12.5% of total rat brain volume [35]. The control groups consisted of sham surgery or naïve animals.

All other experiments involving rats were performed at Banyan Biomarkers with University of Florida IACUC approval. Rats injured by the controlled cortical impact (CCI) model were generated as described [36]. CCI was induced on anesthetized male Sprague-Dawley rats (Harlan). Where indicated, calpain inhibitor SNJ-1945 (100 mg/kg bolus, i.v., in a formulation of PEG 12.5%/EtOH 60%/PBS) was administered immediately after CCI. After 24 h, animals were anesthetized and sacrificed by decapitation. The cortex and hippocampus were removed from both hemispheres, rinsed in ice cold PBS, snap-frozen in liquid nitrogen and stored at −80°C until use.

### Brain Lysates

Human post-mortem brain tissue was purchased from Analytical Biological Services. For western blot analysis, human or rat brain tissue was pulverized to a fine powder with a mortar and pestle set over dry ice. The pulverized brain tissue was then lysed for 4 hours at 4°C in 20 mM Tris-HCl pH 7.4, 5 mM EDTA, 5 mM EGTA, 1% Triton X-100, and 1 mM DTT, and complete protease inhibitor cocktail (Roche) followed by centrifugation at 10,000×g for 10 min at 4°C [36],[37]. Calpain-2 and caspase-3 *in vitro* digestions of rat brain lysates were performed as described [36],[37].

### Manifold Autoantibody Immunoblotting Assay

Samples were subjected to SDS-PAGE on 4–20% Tris-glycine gels and electrotransferred to PVDF membrane. PVDF membranes were then clamped into the Mini-Protean II Multiscreen apparatus (Bio-Rad), and individual lanes were blocked and probed with human sera diluted at 1∶100, unless otherwise noted. Secondary antibodies used were either alkaline phosphatase (AP)-conjugated goat anti-human IgG+IgM or AP-conjugated donkey anti-human IgG diluted 1∶10,000 (Jackson ImmunoResearch). Quantification of autoantibody reactivity on immunoblots was performed via computer-assisted densitometric scanning (Epson 8836XL high-resolution scanner and NIH Image J densitometry software). Autoantibody levels were expressed in arbitrary densitometry units. Statistics were performed using GraphPad Prism 5.0. The Mann-Whitney U test (two tailed) was used to evaluate the difference between two groups, with p<0.05 taken as significant. Spearman correlations were used to explore the relationship between fold change in autoantibody levels and outcome measures.

### Anion-exchange Chromatography and Tandem Mass Spectrometry

The BioLogic Duo-Flow Chromatography System (Bio-Rad) was used. The mono-Q column (GE Healthcare) was connected to a QuadTec UV detector and BioFrac fraction collector. Human cortex protein samples consisting of 5 mg protein were injected. The buffer was ice-cold 20 mM Tris-HCl (pH 8.0) with 1 mM EDTA and 1 mM DTT. Elution was performed with 0.5 M NaCl in buffer at a flow rate of 1 ml per min and monitored at 280 nm. Twenty-six 1 ml fractions were collected. Laemmli sample buffer was added to the YM-10 collection filters prior to centrifugation at 3500 rpm for 3 min.

Excised gel bands were cut into 1 mm cubes, washed in HPLC grade water, destained with 50∶50 100 mM ammonium bicarbonate/acetonitrile until completely colorless and then dehydrated with acetonitrile. The proteins were reduced with 100 μl of 45 mM DTT at 55°C for 30 min. After cooling to room temperature, DTT was replaced with 100 μl of alkylating agent (100 mM iodoacetamide) and incubated in the dark for 30 min. The gels were washed 3 times with 100 μl 50% Acetonitrile/50% 50 mM ammonium bicarbonate and dehydrated with acetonitrile. The dried gel cubes were rehydrated with 15 μl of 12.5 ng/μl sequencing grade trypsin (Promega) and kept on ice for 45 min. Twenty microliters of 50 mM ammonium bicarbonate was added and digestion was performed at 37°C overnight. The tryptic peptide extracts were centrifuged under vacuum until dryness and the residue was reconstituted in 15 μl of water with 0.1% formic acid.

Nanoflow reversed phase chromatography was performed on a Nanoacquity Waters HPLC system (Waters). Samples were loaded via an autosampler first onto a 5 μm Symmetry 180 um×20 mm trap column at 4 μl/min for 10 min and then directed to a 1.7 μM BEH130 C18 100 μm×100 mm column at a flow rate of 250 nl/min. The mobile phase consisted of solvent A (99% water/1% acetonitrile with 0.1% formic acid) and solvent B (75% acetonitrile/25% water with 0.1% formic acid). Separation was achieved using a run time of 111 min. The elution gradient was from 2% to 40% B over 90 min, the followed by 40% to 80% B over 5 min and held for 5 min before returning to the initial mobile phase composition (2% B). Tandem mass spectra were collected on a Thermo LTQ-XL (Thermo-Fisher) using a data dependent acquisition. MS/MS spectra were extracted by Xcalibur version 2.7.0. All MS/MS spectra were analyzed using Sequest (Thermo-Fisher; version SRF v. 5) and X! Tandem (www.thegpm.org; version 2007.01.01.1). Sequest (v. sp3.1.1) was set up to search the trypsin indexed ipi.HUMAN (v3.59) database and X! Tandem was set up to search a subset of the ipi.HUMAN database (v3.59), using a fragment ion mass tolerance of 1.00 Da and a precursor ion tolerance of 2.5 Da. Carbamidomethylation of cysteine was specified as a fixed modification and oxidation of methionine as a variable modification in Sequest and X! Tandem. Peptide identifications using Scaffold (version Scaffold_2_04_00, Proteome Software Inc.) were accepted if they could be established at greater than 95% probability as specified by the Peptide Prophet algorithm, and protein identifications were accepted if they could be established at greater than 99.9% probability and contained at least 2 identified peptides [38]. Using these filtering criteria, Scaffold calculated 0% false positive rate.

### Primary Cerebrocortical and Glia Culture

For all cell culture experiments, one-day old Sprague-Dawley rat pups were euthanized with sodium barbital. Cerebrocortical (CTX) cells harvested from brains were plated on poly-L-lysine coated 12 well culture plates at a density of 4×10^6^ cells/well and maintained as described [36]. Experiments were performed 10–11 days post-plating. MTX was used at 3 nM for 3 h, and EDTA was used at 5 mM for 24 h [39]. Cells were pretreated with either the calpain inhibitor SNJ, or the pan-caspase inhibitor Z-VAD-FMK for 1 h before drug challenges as described [40].

Astrocytes were harvested from rat pup brains, which were placed in Hanks’s Balanced Salt Solution (HBSS; Life Technologies) on ice. The cortex was isolated and stripped of the meninges. Cortices from two pups were minced with fine scissors and then transferred to a 15 ml sterile tube, resuspended in 5 ml cold HBSS, and centrifuged at 200×g for 3 minutes at 4°C. Supernatant was aspirated, the pellet was resuspended in 5 ml 0.5% trypsin, then incubated for 25 minutes in a shaking 37°C water bath. After digestion, the suspension was centrifuged as above, and the pellet was rinsed 3 times with 3 ml HBSS. Next 6 ml astrocyte medium [DMEM containing 10% FBS supplemented with fungizone and 50 μg/mL gentamycin (Life Technologies)] was added and the pellet was triturated to dissociate cells. The cells were then filtered through a 70 μm nylon mesh strainer (Small Parts) into a 50 ml sterile tube. The number of viable cells was counted using a hemocytometer. Cells were plated on 12 well poly-L-lysine-coated tissue culture plates at 9.5×10^4^ cells/well. Cells were incubated at 37°C in a 5% CO_2_ atmosphere. Medium was changed every other day and cells were used after 2–3 weeks.

### HEK293 Cell Culture and Transfections

Human embryonic kidney 293 (HEK293) cells were obtained from ATCC and maintained in high glucose DMEM (Invitrogen) supplemented with 10% FBS and 50 μg/mL gentamycin in 5% CO2 at 37°C. At 50% confluence, cells were transfected as per the manufacturer’s instructions with a 1∶5 ratio of DNA:METAFECTENE™ (Biontex, Martinsried, Germany, cat no. T020). eGFP-N2 expression vector was obtained from Clontech. Human full length GFAP cDNA was obtained from OriGene (SC118873). PCR was used to amplify cDNA corresponding to truncated GFAP (38 kDa) using the primers F: 5′ AAAGAATTCACCACCGAAACGATGGCTGGCTTCAAGGAGACCC and R: 5′ TTTTCTAGATCAGGTCTGCACGGGA ATGGTG and full length GFAP cDNA as template. The resulting 971 bp fragment was then subcloned into the pEF-IRES-puro6 vector using the unique restriction sites Eco RI and Xba I.

### Immunohistochemistry

Animals were anesthetized with a lethal dose of beuthanasia-D solution and transcardially perfused with 4% paraformaldehyde. Whole brains were removed, processed, embedded in paraffin, and cut into 4–6 μm sections. After de-parafinization, slides were incubated for 10 min at 95°C in Trilogy solution (Cell Marque), blocked with 3% hydrogen peroxide, followed by 2% normal goat serum. The sections were incubated with TBI or normal human serum overnight at 4°C, then incubated with goat anti-human HRP (Abcam) diluted in 2% goat serum. Staining was visualized with 3,3′-diaminobenzidine (DAB; Dako). The sections were counterstained with hematoxylin (Dako). Sections were finally washed with PBS, mounted, air-dried and cover slipped with Aquamount (Dako). The slides were scanned and examined using the Aperio ScanScope GL system at 20 x and ScanScope software. For immunofluorescence experiments, sections were incubated with anti-GFAP Alexa Fluor 555 conjugate (Cell Signaling) and either normal or TBI human serum overnight at 4°C, followed by goat anti-human Alexa Fluor 488 (Life Technologies) diluted in 2% goat serum. The sections were washed with PBS, mounted, air-dried and cover slipped with FluoroGel (GenTex). Staining was imaged with a Zeiss fluorescence microscope.

### Immunocytochemistry

Rat primary astrocytes were cultured on CC2 glass chamber slides (Nunc). Astrocytes were rinsed 2x with DMEM, and fixed with 4% paraformaldehyde for 5 minutes. Cells were washed with 50 mM Tris pH 7.4, 150 mM NaCl (TBS), then permeabilized with 0.1% Triton X-100 for 5 minutes. After washing, cells were digested with 1 μg/ml rat calpain-2 in TBS, 10 mM CaCl_2_, 20 mM DTT for 30 min. Undigested controls were treated in parallel with TBS, 10 mM EGTA, 20 mM DTT (no enzyme). Cells were washed with TBS, 10 mM EGTA, and post-fixed with 4% paraformaldehyde for 5 minutes. Cells were blocked with 2% goat serum in PBS for 30 minutes. GFAP antibody (Abcam), human normal serum, or TBI sera were diluted in blocking solution, added to cells, and incubated overnight at 4°C. The next day, cells were washed 3 times with PBS. Fluorochrome-conjugated secondary antibodies (Jackson ImmunoResearch) and Hoechst (10 μg/ml; Life Technologies), diluted in blocking solution, were added to cells (30 minutes). Cells were washed with PBS, mounted using ProLong Antifade (Life Technologies), and imaged with an Olympus 1X81-DSU spinning disk confocal microscope.

## Results

### Circulating Brain-specific Autoantibodies Developed after TBI in Humans

**Figure 1:**
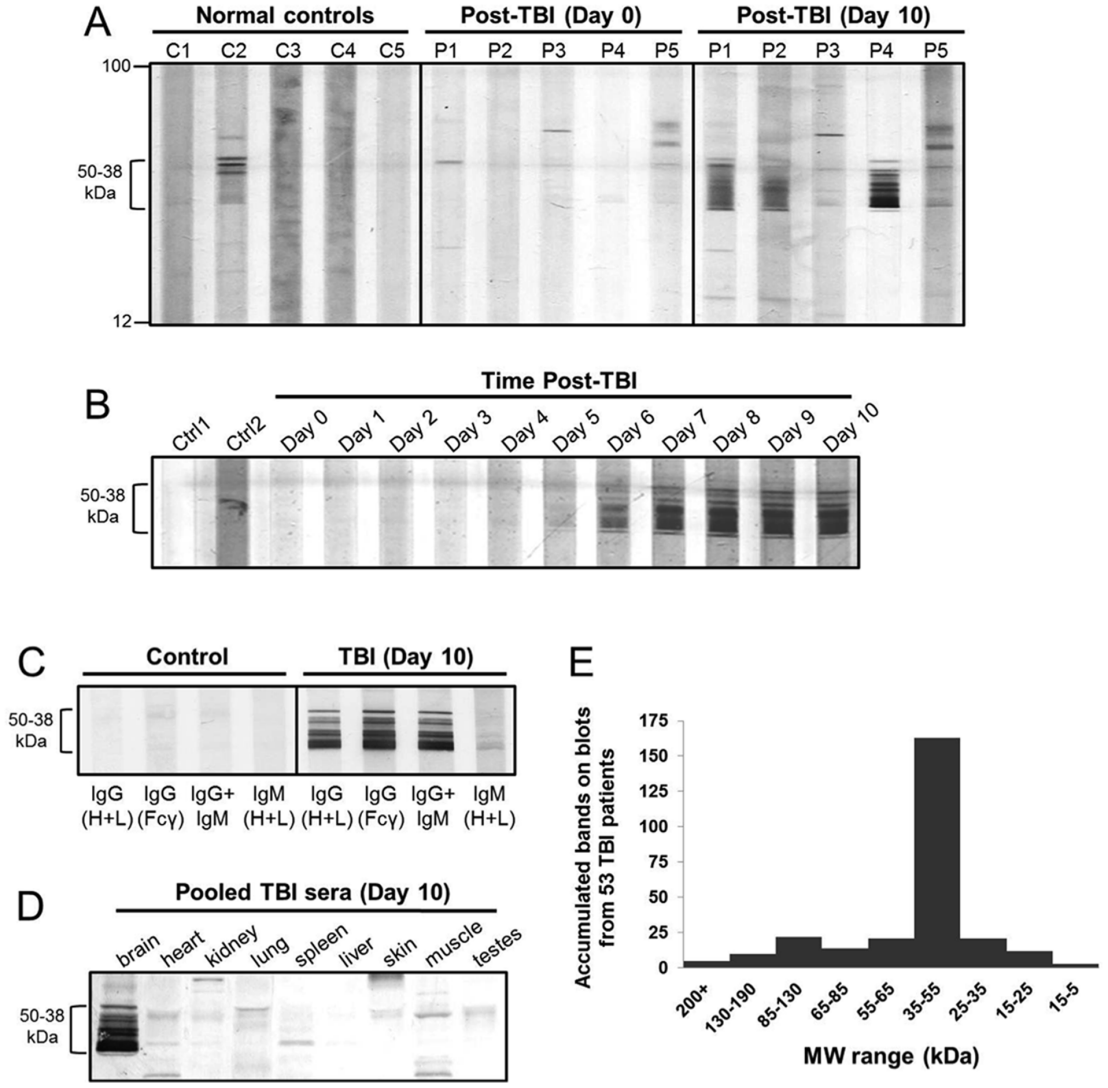
Human severe TBI patients developed circulating IgG autoantibodies against brain proteins within the 50-38 kDa range after injury. **A.** An immunoblot of human brain lysate probed with sera from 5 normal controls (C1–C5) and 5 TBI patients (P1–P5), the latter at two time points post injury (Day 1 and Day 10). Sera were used at 1∶100 except TBI P4, which was 1∶2000. **B.** An immunoblot of human brain lysate probed with sera from 2 normal controls (Ctrl), or daily serum samples from a strongly immunoreactive TBI patient (Days 0–10 post TBI). **C.** An immunoblot of human brain lysate probed with control or TBI serum (Day 9), and then developed with secondary antibodies against human IgG or IgM. **D.** Lysates from a panel of human organs probed with pooled TBI sera (Day 10; n = 4). **E.** Sera from 53 TBI patients (Day 4–10) blotted individually against human brain lysate. Lanes on blots were arbitrarily subdivided into 5 kDa increments using vision works LS image acquisition software. The total number of bands in all lanes were counted, summed and plotted according to MW.

To screen for brain-directed autoantibodies, serum samples from severe human TBI patients (n = 53) or non-head injured normal controls were screened against human post-mortem brain lysate by western blotting. A manifold immunoblotting apparatus was used to screen up to 16 serum samples per blot in parallel. To determine whether brain-directed autoantibodies were pre-existing or triggered by injury, serum samples drawn during the first 24 hours after injury (Day 0) or on the 10th day after injury (Day 10) initially from five subjects were compared individually to normal controls (n = 5). Although signal intensity varied among patients, Day 10 TBI sera in 3 out of five subjects (P1, P2, P4) recognized a cluster of protein bands with molecular weights (MW) between 38 and 50 kDa, based on comparison to MW markers (**Fig. 1A**). Day 0 sera from the same TBI patients or from normal individuals showed relatively little immunoreactivity in the 38–50 kDa region (**Fig. 1A**). The origin of the bands in this region for normal subject in lane C2 is unknown. We had no prior medical histories for the normal individuals or whether they had previously experienced TBI. To explore the timing of autoantibody development post TBI further, the time course from one strongly reacting patient is shown (**Fig. 1B** **)**. Importantly, autoantibody reactivity bands were only observed intensely from Day 6 to Day 10. In all, 9 of the 12 patients examined in this way showed increasing immunoreactivity toward the 38–50 kDa autoantigen cluster with time post injury, with variable signal intensities. We then expanded our autoantibody examination to 12 TBI patients up to Day 10. In 9 out of 12 TBI patients, autoantibodies against the 38–50 kDa cluster were first detectable from 4–7 days after injury, with levels rising thereafter up to Day 10. All 9 of these patients showed immunoreactivity toward the 38–50 kDa autoantigen by Day 7 (data not shown). Of the remaining 3 patients, 2/12 showed no signals, and 1/12 was positive toward the 38–50 kDa autoantigen at all time points examined. We do not know whether this particular positive patient had previously experienced TBI. These results indicated that brain injury triggered the development of circulating autoantibodies against the 38–50 kDa brain autoantigen.

To determine whether TBI autoantibodies represented an immediate and temporary (IgM-based) or a sustained (IgG-based) immunoresponse, autoantibody subclass was examined. Results revealed that TBI autoantibodies were predominantly IgG (n = 3, **Fig. 1C**). To test for brain-specificity of the 38–50 kDa autoantigen, Day 10 TBI sera pooled from 4 patients were also screened against protein lysates made from a panel of human organs. The TBI-associated 38–50 kDa autoantigen cluster was detected in the brain, but not in the 8 other organs tested (**Fig. 1D**).

To extend these findings, the latest serum sample available from each of 53 severe TBI subjects (Day 4–10 post injury) was screened individually against human brain lysate using the manifold blotting assay. **Fig. 1** **E** shows a quantitative analysis of the distribution of TBI-associated immunoreactive bands from these TBI patients. High-resolution images of scanned immunoblots from each patient were subdivided into the indicated molecular weight ranges in 5 kDa increments. The total number of all immunoreactive bands within each increment was then counted and plotted according to MW. Collectively, TBI patients showed the highest number of immunoreactive bands against brain antigens in the 35–55 kDa range (**Fig. 1E**). Although the MW of the brain autoantigen cluster measured by the software (35–55 K) differed slightly from the MW ladder (38–50 K), this assessment reveals that TBI patients showed a dominant immune response against unknown brain protein cluster in the 38–50 K range.

### The 50-38 kDa TBI Autoantigen was GFAP and its Breakdown Products

**Figure 2:**
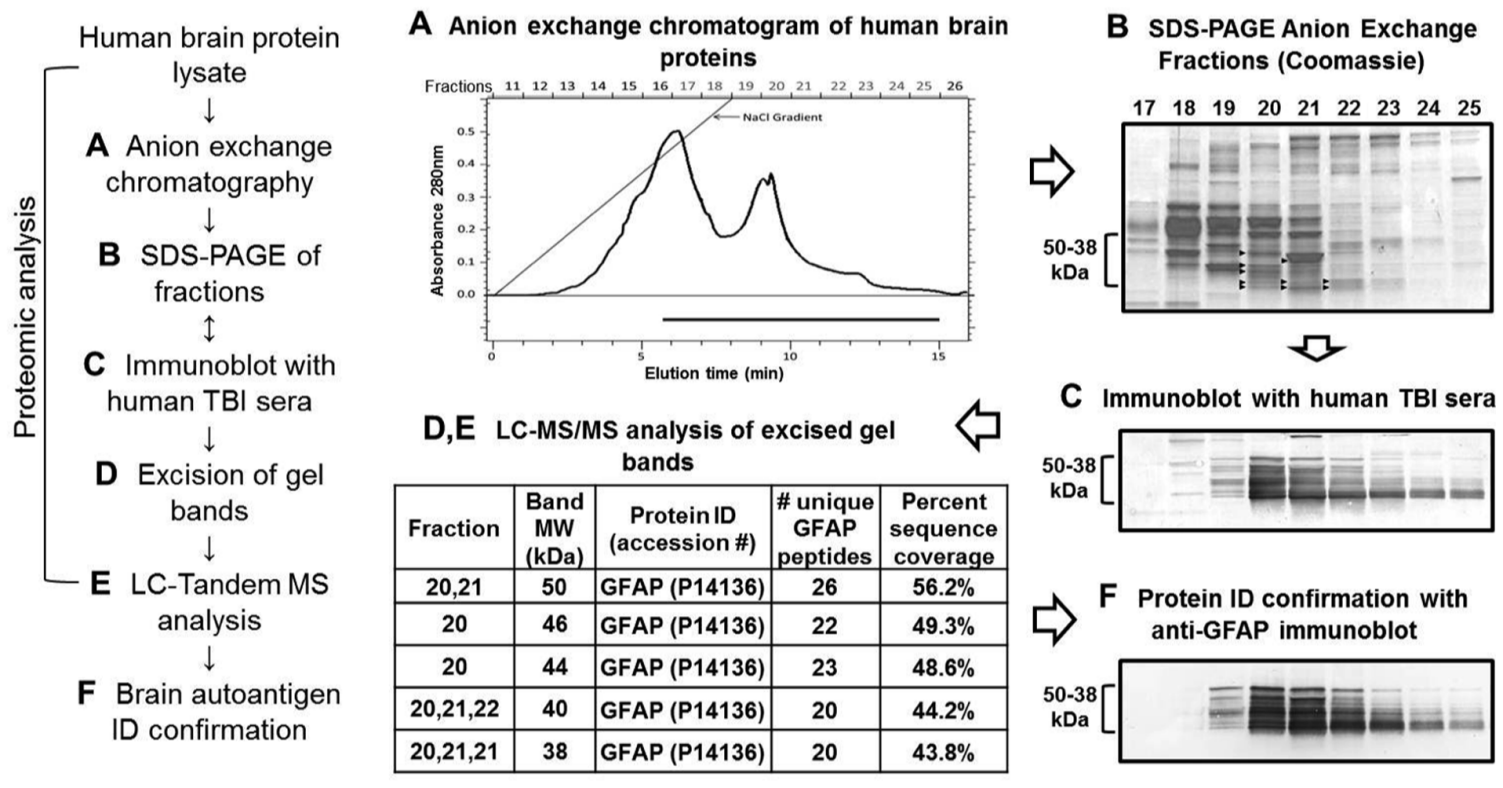
A proteomics approach identified GFAP as the predominant TBI-associated autoantigen. Workflow of the proteomic analysis is shown at left. **A.** Human brain lysate was subjected to anion-based chromatographic separation. A graph of absorbance at 280 nm of fractions 11–26 with elution time is shown. **B.** Fractions were subjected to SDS-PAGE separation, and gels were stained with Coomassie blue to visualize proteins (fractions 17–25 are shown). **C.** Duplicate gels were blotted and probed with pooled TBI sera (Day 10; n = 4). **D, E.** The Coomassie gels in B were overlaid by TBI blots in C, immunoreactive bands from fractions 20–22 were excised for LC-MS/MS analysis (arrowheads), and results are shown in the table. **F.** A duplicate blot to that in C was probed with anti-GFAP antibody (Abcam) to confirm that GFAP was the autoantigen.

Clustering of the autoantigen bands suggested they might be derived from a single protein. To reveal the identity of the autoantigen cluster, a neuroproteomics approach involving liquid chromatography separation and tandem mass spectrometry (MS) was used. Human brain lysate proteins were first fractionated on a strong anion exchange column (**Fig. 2A**), separated by SDS-PAGE (**Fig. 2B**), and then the autoantigen was visualized on blots using pooled Day 10 TBI sera from 4 patients (**Fig. 2C**). Multiple bands (of molecular weight 50 to 38 kDa) from fractions 20–22 were excised from gels, and subjected to LC-MS/MS analysis (**Fig. 2D, E**). Glial fibrillary acidic protein (GFAP) was readily identified in several bands by multiple unique peptides, with high sequence coverage (43.8–56.2%; **Fig. 2D, E**). To confirm that GFAP was the autoantigen, a duplicate blot of fractionated human brain lysate was probed with an anti-GFAP antibody (**Fig. 2F**). The GFAP blot showed an almost identical banding pattern to that generated with human TBI sera (**Fig. 2F**). Since the calculated molecular weight of GFAP should be 50 kDa, based on the multiple banding presentation (**Figure 1C, D**), and the post-mortem nature of the human brain lysate we used as human brain protein source, we speculate that the lower molecular weight bands (46, 44, 40 and 38 kDa) (**Figure 1E, F**) are likely to be breakdown products (BDP) of GFAP.

To confirm GFAP as the dominant autoantigen following TBI, an antigen competition experiment was conducted (**Supplementary Fig. S1**). Prior to probing the blot of human brain lysate, GFAP primary antibody or Day 10 TBI serum from each of 3 TBI patients was preincubated in the absence or presence of 10 μg purified GFAP or Tau. Tau was selected due to its similar MW to GFAP, multiple isoforms, brain specificity, brain enrichment, and association with neuronal damage. For the GFAP primary antibody, preincubation with GFAP prevented the antibody from binding to GFAP bands on the blot (**Supplementary Fig. S1**). Similarly, for three TBI patients tested individually, preincubating Day 10 TBI serum samples with GFAP protein effectively blocked recognition of the brain autoantigen cluster, while Tau did not (**Supplementary Fig. S1**). These data revealed that the 38–50 kDa brain autoantigen was in fact GFAP.

### Calpain Removed GFAP Termini to Generate a 38 kDa GFAP-BDP

**Figure 3:**
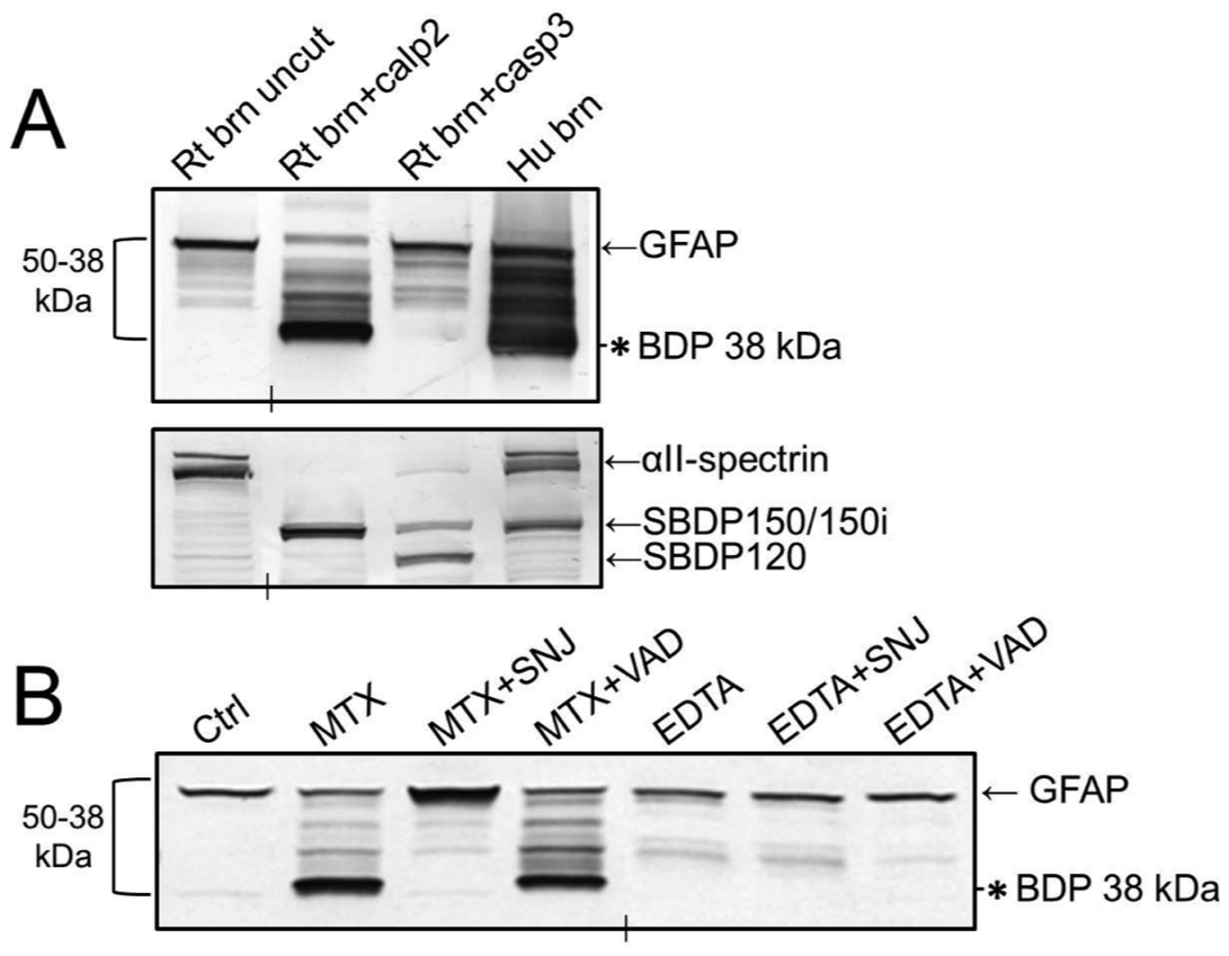
Calpain-associated GFAP breakdown occurred *in vitro* and in primary rat cortical cultures. **A.** Rat brain (Rt brn) lysate was subjected to calpain-2 or caspase-3 digestion *in vitro*, blotted, and probed with antibodies against GFAP (Abcam; top panel) or αII-spectrin (Enzo; bottom panel). Calpain-2 produced a banding pattern similar to the human brain lysate, while caspase-3 did not. Enzymes produced their characteristic spectrin BDP patterns (SBDP150 for calpain, SBDP150i and SBDP120 for caspase). **B.** Rat CTX mixed cultures were untreated (Cntl) or subjected to the indicated drug treatments, then blotted and probed with GFAP antibody (Abcam). The calpain activator maitotoxin (MTX), but not the caspase activator EDTA, induced strong GFAP breakdown to the 38 kDa GFAP band. The calpain inhibitor SNJ1945 (SNJ), but not the caspase inhibitor Z-VAD-fmk (VAD), blocked GFAP breakdown by MTX. In A and B, vertical hash marks indicate where intervening lanes were removed. An asterisk marks the 38 kDa GFAP band throughout.

When human brain lysate was probed with either anti-GFAP antibody or TBI sera (**Figs. 1** **–** **2**), besides intact GFAP at 50 kDa, a range of intermediate bands down to 38 kDa was also observed. This banding pattern suggested that GFAP had been subjected to post-mortem proteolysis. Our previous reports state that GFAP in fact is vulnerable to proteolytic processing linked to calpain activation [41],[42]. As reported previously, GFAP is proteolyzed by the cell death protease calpain *in vitro*, and a 38 kDa GFAP breakdown product (GFAP-BDP) accumulated as the limit fragment. TBI results in activation of calpains as well as caspases, which leads to necrotic and/or apoptotic cell death [11],[43]–[45]. To test whether GFAP can be cleaved by caspase, *in vitro* digestions of rat brain lysate (containing GFAP) were performed. Immunoblot results showed that calpain-2 cleaved rat brain GFAP to 38 kDa as expected, while caspase-3 did not (**Fig. 3A**). We did observe a modest increase of the 42–43 kDa bands upon caspase-3 digestion. In the same lysates, calpain cleaved αII-spectrin to SBDP150, and caspase-3 to SBDP150i and SBDP120, respectively (**Fig. 3A**), demonstrating that the proteases were active. These results indicated that GFAP is degraded readily by calpain, but more limitedly by caspase-3, *in vitro*.

To test GFAP’s sensitivity to these proteases within cultured cells, calpain or caspase activation was induced in primary rat mixed cortical cultures by treating them with the necrosis-inducing, potent calcium channel opener maitotoxin (MTX), or with the apoptosis-inducing calcium chelator EDTA [39], MTX treatment alone induced robust breakdown of GFAP to a 38 kDa BDP (**Fig. 3B**). Appearance of a 38 kDa GFAP-BDP after MTX treatment was completely inhibited by addition of the calpain inhibitor SNJ1945 (SNJ) to the culture medium, but not by the caspase inhibitor Z-VAD-fmk (**Fig. 3B**). EDTA induced low levels of Z-VAD-sensitive GFAP-BDPs at ∼42–43 kDa, but not the 38 kDa fragment (**Fig. 3B**).

**Figure 4:**
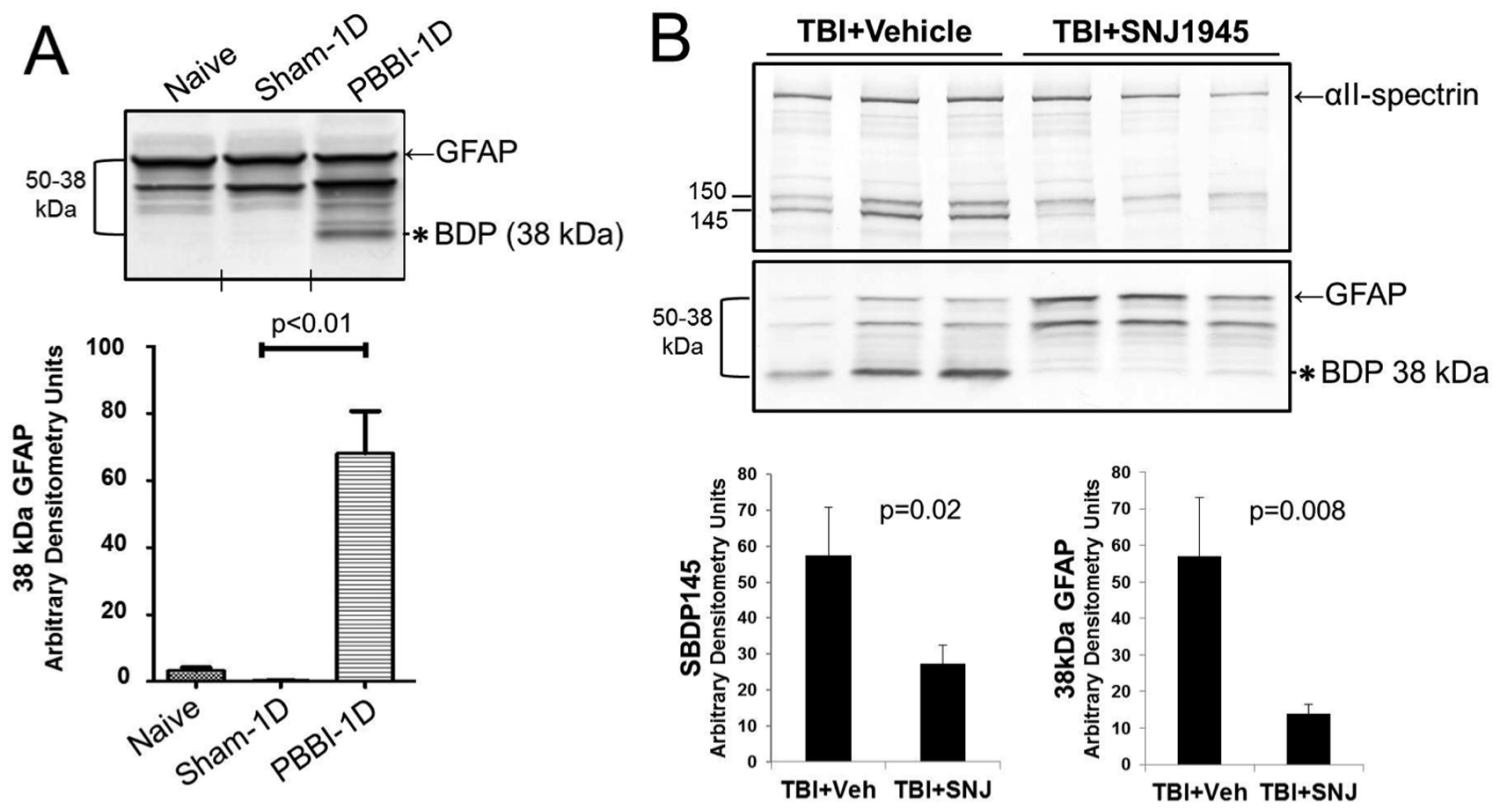
Calpain-associated GFAP breakdown to 38 kDa occurred *in vivo*. **A.** Rats experienced penetrating ballistic-like brain injury (PBBI). Control (naïve, sham) or PBBI brain lysates at 1 day (1D) after injury were immunoblotted and probed with GFAP antibody (Banyan). The 38 kDa GFAP bands were quantified by densitometry. This band was significantly increased compared to controls post PBBI (mean ± SEM; n = 5 per group). Vertical hash marks indicate where intervening lanes were removed. **B.** Rats were injured by controlled cortical impact (CCI), and injected either with vehicle (n = 3) or the calpain inhibitor SNJ1945 (n = 5). Then brain lysates were immunoblotted and probed with antibodies against αII-spectrin (Enzo) or GFAP (Banyan). Quantification of the SBDP145 and 38 kDa GFAP bands showed that SNJ1945 significantly inhibited formation of SBDP145 and 38 kDa GFAP (mean ± SEM). An asterisk marks the 38 kDa GFAP band throughout.

Next GFAP breakdown after TBI was examined *in vivo* using two rat TBI models. First, a rat model of experimental penetrating ballistic-like brain injury (PBBI) was used [1]. Calpain activation is an established feature of rat PBBI [41]. Cortex was collected from the injured region of PBBI rats at 1 day post-injury, or from naïve and sham rats as controls, and examined for GFAP by western blot. Rat cortex at 1 day post-PBBI showed significantly increased GFAP breakdown to a 44 kDa and a 38 kDa fragment. In particular, the 38 kDa band was not observed in the controls (**Fig. 4A**). Similarly, cortex was examined from rats subjected to controlled cortical impact (CCI) injury [46]. In this experiment, CCI-injured rats were treated with calpain inhibitor SNJ or its vehicle immediately after CCI. Then 1 day later, cortex was examined by immunoblotting for αII-spectrin and GFAP. In vehicle-treated cortex, αII-spectrin showed breakdown to the calpain-specific SBDP145 fragment, while in SNJ-treated cortex, SBDP145 was significantly reduced (**Fig. 4B**). Likewise, GFAP breakdown to 38 kDa was present in vehicle-treated injured cortex, but was significantly reduced in SNJ-treated cortex (**Fig. 4B**). These results are consistent with GFAP breakdown associated with calpain after TBI in animal models.

**Figure 5:**
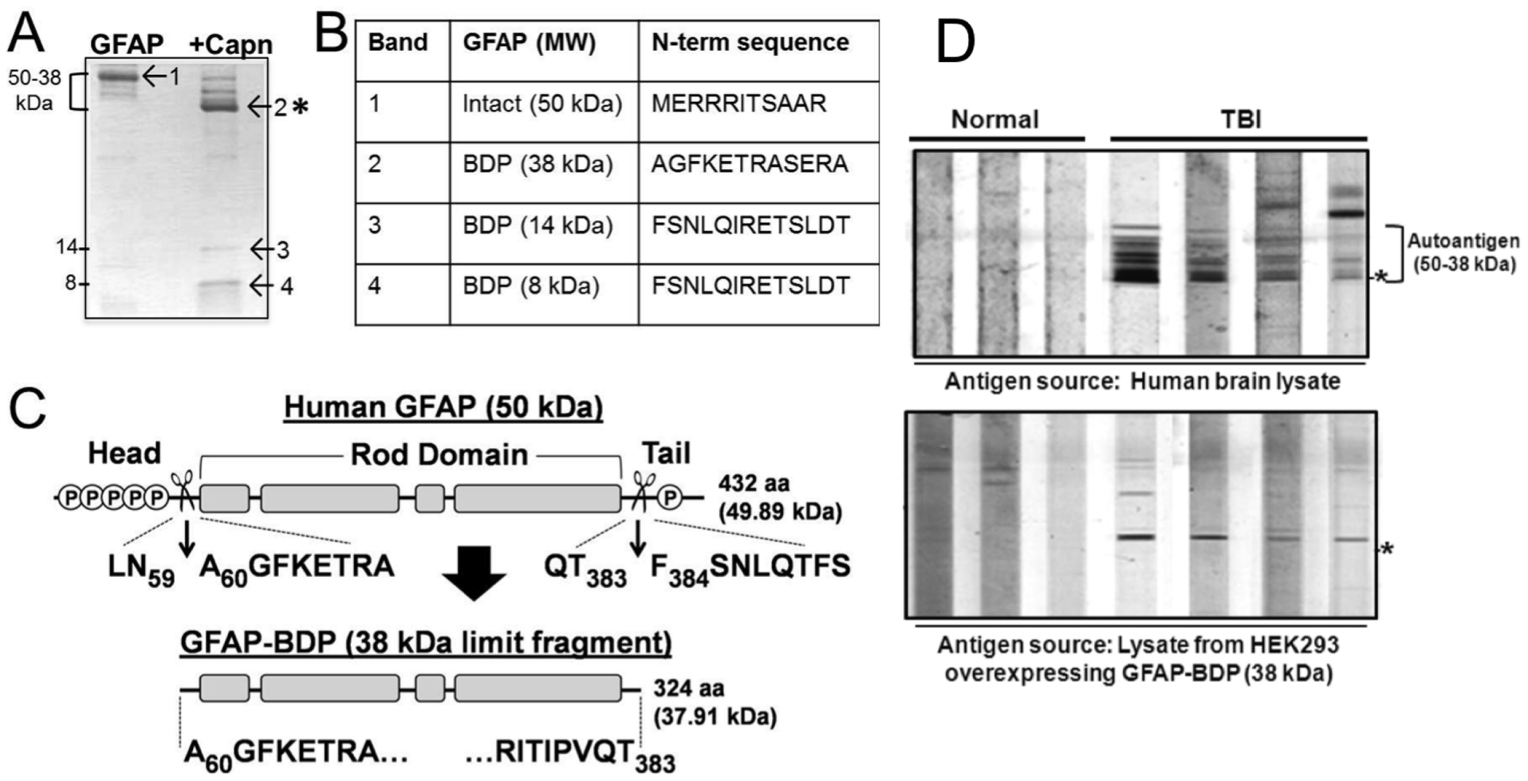
Calpain removed the termini of GFAP to generate the 38-BDP. **A.** Human GFAP was undigested or digested with calpain (Capn), resolved on SDS-PAGE, stained with Coomassie blue R-250, and the indicated bands were excised. An asterisk marks the 38 kDa GFAP band. **B.** The N-terminal sequence of each band is shown in the table. **C.** Calpain cleavage sites (scissors) near the N- and C-termini of GFAP are shown in a schematic drawing of the GFAP. Subdomains (head, rod, tail) and identified/putative phosphorylation sites (P) are indicated, and the predicted 38 kDa calpain fragment is shown. **D.** Three control and four post-TBI human serum samples (Day 4–10) were probed against human brain lysate (top panel) and cell lysate from HEK cells overexpressing the 38 kDa GFAP-BDP (lower panel).

Because results supported GFAP being a calpain target post-TBI, major calpain cleavage sites in GFAP were mapped. Recombinant human GFAP was digested *in vitro* with calpain-2 and resolved by SDS-PAGE (**Fig. 5A**). N-terminal Edman sequencing of GFAP-BDPs revealed that calpain cut GFAP after amino acids N-59 and T-383 (**Fig. 5B**). This result is consistent with previously reported N-terminal cleavage site (after N-59) of GFAP [47]. Cleavage at both sites would remove the head and tail domains of GFAP, yielding a BDP with a calculated MW of 37.91 kDa (**Fig. 5B**). This predicted size is consistent with the 38 kDa GFAP-BDP observed in this study.

To examine whether human TBI autoantibodies prefer GFAP and its BDPs, we overexpressed a cDNA encoding the 38 kDa calpain generated GFAP-BDP in HEK293 cells by transient transfection. As shown in **Fig. 5D** with 4 representative human TBI serum samples (day 4–10), their autoantibodies recognize HEK293-overexpressed 38 kDa GFAP-BDP, as well as in the post-mortem human brain lysate.

### TBI Autoantibodies Recognized GFAP in Injured Rat Brain and Cultured Rat Astrocytes

**Figure 6:**
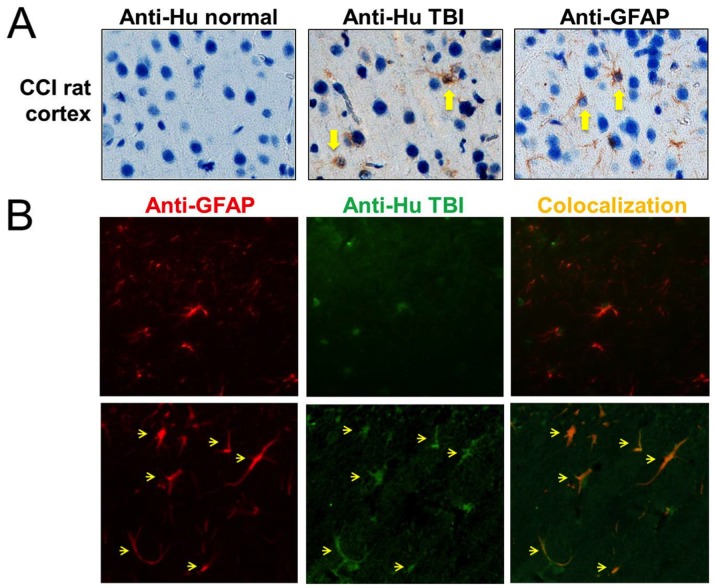
TBI autoantibodies labeled GFAP in injured rat brain. **A.** In sections of cortex from rats injured by CCI, anti-GFAP antibody (Cell Signaling) or human Day 10 TBI serum showed similar patterns of immunoreactivity (brown), while human normal serum did not. Nuclei are blue. **B**. Sections of hippocampus from naïve or CCI-injured rats were incubated with anti-GFAP antibody (Cell Signaling) plus human Day 10 TBI serum, and bound antibodies were visualized with fluorescent secondary antibodies (anti-GFAP in red, TBI serum in green). Image contrast was adjusted in the panels individually to best display the fluorescence. In naïve hippocampus, TBI autoantibodies showed little binding, while in CCI hippocampus, anti-GFAP antibody and TBI autoantibodies showed increased immunostaining, which partially colocalized (orange, arrows).

To determine whether TBI autoantibodies could recognize native GFAP in the brain, immunohistochemistry (IHC) was performed. First, cortical sections from rats that had undergone CCI a day previously were probed with human normal serum, human Day 10 post-TBI serum, or anti-GFAP antibody. Normal serum showed little immunoreactivity, while human TBI serum and GFAP antibody detected cortical cells with the characteristic star-like shape of astrocytes (**Fig. 6A**). Next human TBI serum and GFAP antibody were co-incubated on 5 μm sections of rat hippocampus, and visualized by double immunofluorescence (**Fig. 6B**). In such thin sections, immunofluorescence provides a reasonable measure of colocalization without the need for confocal microscopy. Normal serum showed little immunoreactivity in either control or CCI rat hippocampus (not shown). Human TBI autoantibodies produced minor staining of naive rat hippocampus, which did not overlap with GFAP staining (**Fig. 6B**). By contrast, in the injured hippocampus, TBI autoantibody immunostaining was elevated and partially colocalized with GFAP (**Fig. 6B**). The fact that the overlap between TBI sera and anti-GFAP antibody is not perfectly aligned suggests that there are other autoantigens being recognized by the TBI sera. In any case, these data indicated that TBI autoantibodies were able to recognize GFAP or GFAP-BDP *in vivo* after brain injury.

**Figure 7:**
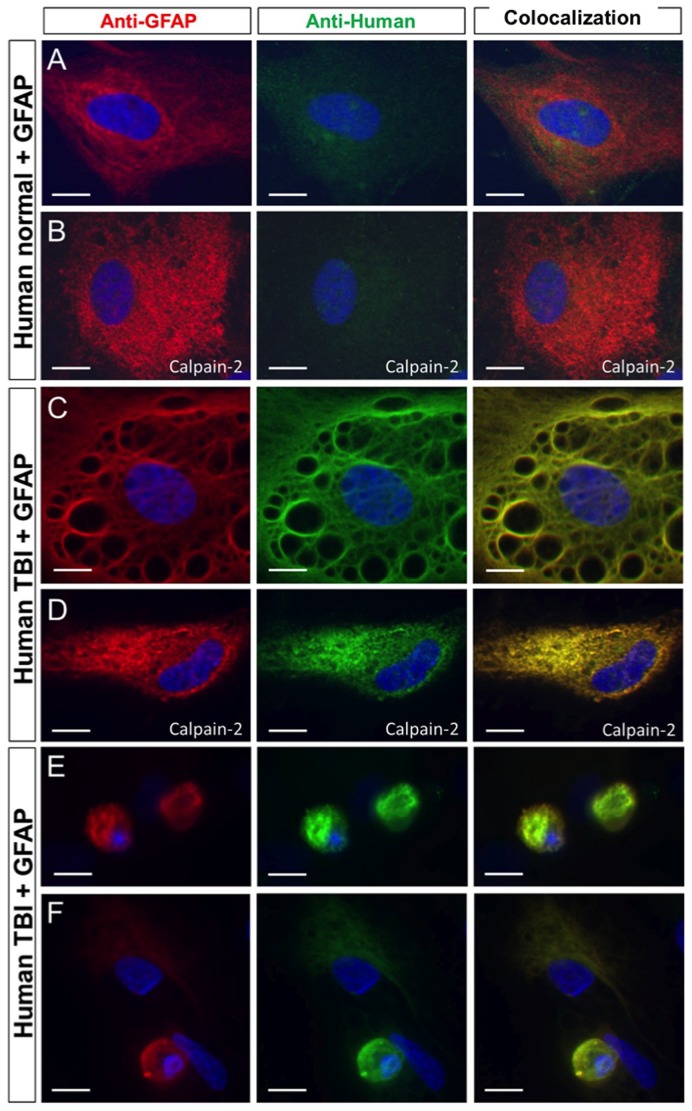
TBI autoantibodies colocalized with GFAP in primary rat astrocytes. Single confocal images (A–E) and flattened z-stacks (F) are shown of astrocytes stained with anti-GFAP antibody (red), human pooled TBI sera (green, Day 9–10, n = 3) or human normal control serum (green). Nuclei are blue. **A.** GFAP antibody stained GFAP fibrils in primary rat astrocytes (left), while human control serum showed diffuse green minor staining (middle) not colocalizing with GFAP (right). **B.** Astrocytes digested with calpain-2 after fixation labeled with GFAP (left) but not normal human serum (middle) as in A. **C.** Immunostaining by GFAP antibody (left) and human TBI sera (middle) strongly colocalized, as evidenced by yellow color (right). **D.** After calpain digestion, staining by the GFAP antibody (left) and human TBI sera (middle) colocalized (right) as in C, without changes in signal intensities. **E.** GFAP antibody (left) and human TBI sera (middle) brightly co-stained (right) rounded cells with condensed nuclear material. **F.** Flattened z-stacks of multiple confocal images are shown, to illustrate the greater staining intensities of GFAP (left) and TBI sera (middle) in a rounded cell compared to healthy cells. Exposure times were shorter in E, F compared to A–D. Scale bars 10 microns.

Next, the ability of TBI autoantibodies to bind GFAP in primary rat astrocytes was examined. This was done for two purposes: to test rigorously whether TBI autoantibodies bind to GFAP in astrocytes, and to determine whether calpain cleavage of GFAP affected the intensity of TBI autoantibody staining. After fixation, primary astrocyte cultures were either undigested or digested with calpain-2, then double-stained with anti-GFAP antibody plus either human normal serum or pooled Day 9–10 TBI sera (n = 3). Immunostaining was then imaged by confocal microscopy. In undigested cultures, GFAP antibody stained GFAP in astrocytes with a filamentous pattern (red; **Fig. 7A** **, left**). Normal human serum showed only minor staining (green; **Fig. 7A** **, middle**) that did not co-localize with GFAP (**Fig. 7A** **, right**). In calpain-digested cells, staining by GFAP antibody acquired a punctate quality from the protease treatment, but otherwise appeared unchanged (**Fig. 7B** **, left**). Staining by human normal serum was also unchanged by calpain (**Fig. 7B**). In contrast, GFAP antibody (red; **Fig. 7C** **, left**), and pooled TBI sera (green; **Fig. 7C** **, middle**) showed strong and striking colocalization (yellow; **Fig. 7C** **, right**) in undigested rat astrocytes. TBI sera produced additional minor staining that did not colocalize with GFAP. The staining intensities of GFAP antibody and TBI sera did not change after calpain digestion (**Fig. 7D**). This experiment confirmed that TBI autoantibodies were able to recognize GFAP in primary rat astrocytes. It also showed that staining intensity was independent of calpain digestion at least for the 3 TBI patient serum samples tested.

Interestingly, in all cultures of rat astrocytes, small, rounded cells were observed. These rounded cells co-stained brightly with GFAP and TBI autoantibodies (**Fig. 7E,F**). Because staining was so intense, exposure times for rounded cells (**Fig. 7E,F**) were reduced compared to cells that were spread out on the substratum (**Fig. 7A–D**). Single confocal images are shown in **Fig. 7E**. Flattened z-stacks of multiple images are displayed in **Fig. 7F**, in order to show the intensity of co-staining in rounded cells compared to surrounding healthy cells. Calpain digestion had no effect on the staining of rounded cells, and rounded cells did not stain with human normal serum (result not shown). We speculate that these are dying dead astrocytes.

### Effects of Human TBI Serum Anti-GFAP Autoantibody Against Primary Rat Glial Cells

**Figure 8:**
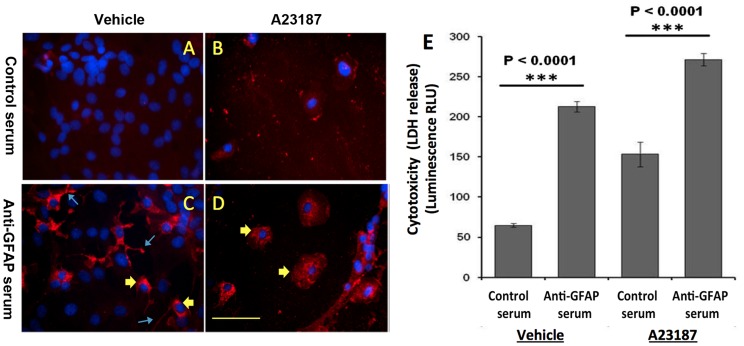
Effects of human TBI serum anti-GFAP autoantibody primary rat glial cells. (**A–E**) Primary rat glia cells were plated on cover slip and cell chamber and incubated in media that included 1/50 serum collected from either human TBI serum with no detectable anti-GFAP autoantibody (Control serum) (**A,B**) or human TBI serum with strong anti-GFAP autoantibody (**C,D**). Cells were treated with either vehicle (DMSO) (**A,C**) or 20 μM A23187 (calcium ionophore) (**B,D**) for 24 hours, After the 24 h incubation, media was removed and the cells were fixed, stained with Hoechst and developed with anti-human IgG secondary antibody, Scale bars 10 microns. Yellow arrowheads indicate glial cell body staining with IgG while blue arrows indicate glial processes staining. (**E**) To test of effects of glial health, we used pooled 4 control samples and 9 TBI samples with strong autoantibody response. Cells were co-treated with either vehicle (DMSO) or 20 μM A23187 for 24 hours. Cell death (by LDH release) was measured using CytoTox-Glo™ Cytotoxicity Assay. Luminescence was read and media from each of the different conditions was used to measure the background, which was subtracted from the individual readings. * (P<0.0001) Represents significant increase of glial death in cells treated with TBI serum as compared to cells treated with control serum (control or with A23187, respectively), using an ANOVA analysis with Bonferroni multiple comparisons.

We also examined the effects of anti-GFAP autoantibodies from human TBI serum on glial cells in culture (**Figure 8**). Either human TBI serum with no detectable anti-GFAP autoantibody (control serum) or human TBI serum with strong anti-GFAP autoantibody was incubated with living primary rat astrocytes in culture. We found that anti-GFAP IgG can gain access to the cytosolic fraction. We observed that even in normal astrocytes, anti-GFAP can label cytosolic fraction (**Figure 8C**) – interestingly, staining is more localized to the cell body and short processes that are more proximal to the cell body of astrocytes – suggesting that anti-GFAP might be inhibiting the assembly and/or function of GFAP, including the support of process extension. When cells were co-treated with calcium ionophore A23187 (20 μM), which activates calpain, glial cell bodies were even more prominently and diffusedly labeled with anti-GFAP autoserum (**Figure 8D**). In contrast, control human serum (absent anti-GFAP antibodies) did not show significant staining under the same conditions (**Figure 8** **A,B**). We also found similar pattern of staining in live glial cells with purified anti-GFAP polyclonal antibody (data not shown).

In addition, we also found that anti-GFAP autoserum induces glial cell injury as is reflected by cytosolic LDH –release (**Figure 8E**). Again A23187 treatment also injured glial cells and increased LDH release. However, the presence of anti-GFAP autoserum during A23187 challenge further exacerbates the LDH release. These effects are not observed with serum that lacks anti-GFAP autoantibody (**Figure 8E**).

### Clinical Relevance of Post-TBI Autoantibodies towards GFAP and GFAP-BDP

**Figure 9:**
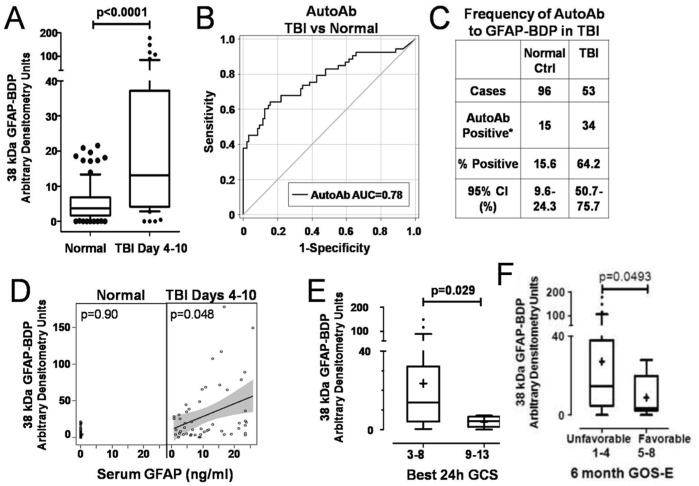
Clinical analyses of GFAP autoantibodies in severe TBI and control subjects. (**A**) Day 4–10 serum GFAP-BDP (38 kDa) autoantibody levels from severe TBI patients were semi-quantified and found to be significantly higher than those from normal controls. (**B**) Receiver Operating characteristic (ROC) curve plotting serum autoantibody levels to 38 kDa GFAP-BDP revealed that autoantibody level was a good discriminator between TBI subjects and healthy normal controls with AUC = 0.78. The average serum autoantibody levels at a cutoff of >8.49 arbitrary unit has a 64% sensitivity, 85% specificity, to distinguish between TBI and controls. (95% CI 0.70–0.87. AUC = area under receiver operating characteristic curve). (**C**) Frequency of autoab against 38 kDa GFAP-BDP in TBI serum (Day 4–10; n = 53) as compared to normal controls (n = 96). *Autoantibody positive represents a discrete immunoreactivity band signal of at least 1.50 densitometric units after background subtraction. (**D**) GFAP-BDP autoantibody correlated significantly to serum GFAP levels at 24 h post-TBI measured by sandwich ELISA. (**E**) A significant correlation between the best GCS score (index of severity) during the first 24 h after injury and GFAP-BDP autoantibody levels (GCS 3–8 are severe, GCS 9–13 moderate). (**F**) GFAP-BDP autoantibody levels at Day 4–10 correlated to outcome measurement GOS-E at 6 months.

The clinical and demographic characteristics of our 53 patients with severe TBI and 96 healthy normal controls are summarized in **Supplementary Table S1**. Sera from all subjects were probed against human brain lysates using the manifold immunoblotting apparatus as in Figure 1. The appearance and intensity of a 38 kDa GFAP-BDP was used as a measure of autoantibody presence in each patient’s serum samples. Consistent with our earlier data (**Fig. 1**), circulating autoantibody against GFAP-BDP was observed in sera of TBI patients 4–10 days after injury (**Fig. 9A**). Using western blots to evaluate the autoantibodies, our patient data is qualitative, rather than quantitative as would be obtained by ELISA. Nevertheless, we were able to evaluate the potential application of GFAP-BDP autoantibodies in diagnosis of subacute TBI, using a receiver operating characteristic (ROC) curve analysis which was performed to discriminate between head injured subjects and controls. The area under the curve (AUC) measures how well a parameter (in this case the level of GFAP-BDP autoantibody) can distinguish between two groups. The AUC was significant at 0.78 (95% confidence interval [CI] = 0.70–0.87, **Fig. 9B**), indicating that autoantibody level did distinguish head-injured from healthy individuals. From the ROC curve, an optimal cutoff value of 8.49 (arbitrary densitometry units) was defined. Based on this value, 64.2% of all TBI patients (34/53) had a positive autoantibody response to GFAP-BDP (day 4–10), compared to only 15.2% in normal controls (15/96) (**Fig. 9C**). Our previous data showed that GFAP and BDP levels return to baseline by 3–4 days after severe TBI [48]. However, we hypothesize that the acute serum level (within first 24 h) should have some degree of correlation to the subsequent development of autoantibody (autoab) response (day 4–10). In fact, we found a significant correlation between the serum level of GFAP protein at 1 day post injury and serum level of GFAP autoantibodies at 4 to 10 days post injury (p = 0.048) (**Fig. 9D**). We also investigated the potential correlations among GFAP-BDP autoantibody levels, injury severity, and prognostic outcome. The best Glasgow Coma Score (GCS) within the first 24 hours after injury was used to classify the severity of TBI. Patients with higher GCS scores of 9–13 (less severe) had significantly lower autoantibody levels (p = 0.029) than patients with lower GCS scores of 3–8 (most severe) **(** **Fig. 9E**). Lastly, serum autoantibody towards 38 kDa levels were significantly higher in patients with unfavorable outcomes as compared to favorable, based on the Glasgow Outcome Score Extended (GOS-E) at 6 months (mean 18.2 versus 4.3, p = 0.0493, Mann-Whitney U test, **Fig. 9F**).

Immunoreactivity and baseline autoantibody levels varied among TBI patients (**Fig. 1**), making absolute quantitative analyses sometimes challenging. Therefore we also attempted a relative quantitative method for anti-GFAP-BDP autoantibody in TBI patients. A determination of the number of TBI patients that developed anti-GFAP autoantibodies post TBI was obtained by computing the fold change in anti-GFAP immunoreactivity over time for each patient. Only those patients having a late serum sample of Day 7 or beyond were selected for this analysis (45 patients). Based on the time course experiment (**Fig. 1B**), Day 7 was determined to be late enough to show an increased autoantibody response, if present. For each patient, the latest available serum sample (Day 7–10) was paired with the earliest available serum sample (Day 0–1), and blots of human brain lysate were probed with each pair. In this way, each patient served as his or her own control, thus bypassing the issue of variability. As a measure of the level of anti-GFAP autoantibodies in each patient, bands at 38 kDa were quantified by densitometry. The ratio of values for the late time point over the early time point was then computed for each patient, to give fold change in autoantibody levels with time post injury. In this analysis, only 45 out of the total 53 TBI subjects fulfilled our relative quantitative method inclusion criteria. The median fold increase of 38 kDa BDP levels was 1.66 times for the 45 TBI patients, with a maximum of 34.7 times, and a minimum of 0 (no immunoreactivity, 1 patient) (**Table 1**). The average increase was 3.77. Thirty of the 45 TBI patients (67%) showed an increase of 1.5 times or more post TBI. For 43/45 patients, clinical outcome data was available. For these 43 patients, GOS-E (Glasgow outcome score at hospital discharge) ranged from 1–4, with 1 indicating death, 2 vegetative state, and 3–4 indicating severe disability. A significant negative correlation between fold change in anti-GFAP-BDP autoantibody levels and GOS-E score at hospital discharge was found (p = 0.03; Spearman r = −0.33) (data not shown). This is consistent with the correlation obtained between absolute anti-GFAP-BDP levels at Day 4–10 and GOSE-E (at 6 months) (**Fig. 9F**).

**Table 1:** Fold change of anti-GFAP-BDP antibody levels at Day 7–10 over day 0–1 among selected severe TBI subjects.

Fold change of GFAP-BDP-38 K	Severe TBI (n = 45)
Minimum	1.00
25% percentile	1.10
Median	1.66
75% percentile	3.44
Maximum	34.70
Mean	3.77
Std, error	0.88

The fold change was calculated as the ratio of 38 kDa band intensities at the later time (Day 7–10) divided by the initial time point (Day 0–1) per patient.

## Discussion

In this study, human severe TBI serum samples were screened in order to identify TBI-associated autoantigens. TBI autoantibodies showed common immunoreactivity against a cluster of bands in the 38–50 kDa region on brain immunoblots, which were identified as GFAP and GFAP-BDPs. Our previous report showed that GFAP protein from 38–50 kDa was present in CSF samples from human severe TBI patients [37]. The present study is consistent with the conclusion that calpain was responsible for cleaving GFAP to 38 kDa. In TBI patient sera, GFAP autoantibody levels increased by 7 days after injury and were of the IgG subtype, suggesting that they could persist much longer. Preliminary data has shown that human post-TBI serum can sustain anti-GFAP autoantibodies for up to 6 months, the latest time point yet examined (unpublished observations). Thus the level of GFAP autoantibodies in serum could potentially serve as the basis of a test for TBI. Each patient could serve as their own control, with an acute serum sample (0–3 days post injury) establishing baseline levels of anti-GFAP immunoreactivity, and a later sample (7–10 days post injury) serving as a test for elevation.

With the methods employed here, out of all the brain proteins represented on immunoblots, TBI serum recognized GFAP most strongly. Using similar methods, rat post-TBI serum did not show apparent immunoreactivity against GFAP (unpublished observations). This could be a result of sensitivity issues, the greater complexity of human disease, temporal differences, or a fundamental species difference. Human TBI serum recognized other bands on brain immunoblots besides GFAP, but at lower intensities (**Fig. 1**). Similarly, when sections of injured rat hippocampus were probed with TBI serum, in addition to labeling GFAP, TBI serum also labeled material that did not co-localize with GFAP (**Fig. 6B**). While these findings indicate that human TBI serum contained immunoreactivity against additional brain autoantigens, the combined data clearly show that TBI patients commonly developed autoantibodies against GFAP, evidenced both by western blotting and rat brain/astrocyte staining.

When primary rat astrocytes were co-stained with GFAP antibody and TBI serum, the intensely stained, rounded cells had morphologies typical of dead/dying cells (**Fig. 7E, F**). Early steps in cell death include retraction of cellular processes and nuclear condensation [39]. Based on morphological criteria, the rounded cells shown in **Fig. 7E, F** were likely dead or dying prior to fixation. Rounded astrocytes stained more intensely with TBI autoantibody than healthy cells. Cellular condensation, and the resulting concentration of GFAP into a reduced volume, was likely responsible for the increased intensity of staining. As a limit fragment of calpain digestion, the 38 kDa GFAP band may be a particularly persistent form of GFAP in dead or dying astrocytes. The intense staining of dead/dying astrocytes suggests that 38 kDa GFAP is stable even in the context of cell death. The important implication for TBI patients is that GFAP-BDPs may persist within degenerating astrocytes in the brain, thus facilitating it becoming a predominant immune target. In addition, we found that anti-GFAP autoantibody can gain entry to live glia cells in culture (**Figure 8** **A–D**). This is consistent with previous work showing that anti-nuclear autoantibodies can also enter cells [49]. We further found that incubation with anti-GFAP autoserum causes cytotoxicity in glial cells (**Figure 8E**). Taken together, these data suggest that the presence of autoantibody to GFAP can be potentially pathogenic during the recovery phase of TBI.

When rat brain sections were co-stained with GFAP antibody and TBI serum, signals from both were stronger in injured rat hippocampus than in naïve hippocampus (**Fig. 6B**). In injured brain, astrocytes respond by proliferating and upregulating GFAP in a process termed reactive gliosis [50]. Condensation of damaged astrocytes or the persistence of 38 kDa GFAP may have also contributed to the increased staining. Alternatively, given that TBI autoantibodies detected the 38 kDa GFAP band most commonly on western blots, it is also possible that TBI autoantibodies preferred to bind to 38 kDa GFAP-BDP over intact GFAP. Not all TBI sera showed such a preference, since the pooled TBI sera in **Fig. 7** detected GFAP in healthy cells (likely intact) with similar intensity to that in calpain-cleaved cells, and calpain digestion did not affect the intensity of staining. The preference of anti-GFAP autoantibodies for various GFAP bands is likely to differ among patients, since each patient’s immunoresponse is unique. This is a subject worth further investigation.

Unidentified brain-directed autoantibodies in human blood can be found in normal subjects [51]. Here, anti-GFAP autoantibodies were present in 15.6% of normal individuals (**Fig. 9C**). It is possible that some control individuals may have had previous unreported/minor head injuries or other undiagnosed neurological issues. It is thus possible that the response seen in normal subjects represents either a low concentration of natural antibodies that cross react with GFAP or specific anti-GFAP autoantibodies due to prior brain injury. When TBI-induced release of additional GFAP and BDPs can produce a boosting effect in such individuals resulting in more production of IgG against GFAP. Anti-GFAP autoantibodies have been preliminarily reported in other human disorders, such as in patients with Alzheimer’s disease or other dementias [18],[19],[52]–[54], stroke [16],[55], diabetes [56], neurological disorders, multiple sclerosis [57],[58], autism [59] and lead-exposed workers [60]. Anti-GFAP antibodies in brain tumors of astrocytic origin (such as glioma and glioblastoma) have also been reported [61]. While these reports reveal that anti-GFAP autoantibodies are associated with a variety of neurological pathologies, the present article represents the first description of anti-GFAP autoantibodies after TBI. We are also the first to identify that post-translationally modified GFAP (GFAP-BDP) is a preferred autoantigen that could contribute to the breakdown of self-tolerance of the immune system following TBI. Indeed, our concept that GFAP-BDP is an immunodominant brain autoantigen following TBI meets two key requirements for the immune system breaking tolerance to self-proteins [62] : (i) Accessibility of the antigen to the immune system: The brain is considered an immune-privileged organ due to the lack of a lymphatic system, and protection by the endothelial blood-brain barrier (BBB). Thus, the release of a brain protein (GFAP-BDP) in elevated quantity and a compromised BBB after TBI would fulfill the first requirement. (ii) Non-homeostatic state of the protein/altered-self (i.e., through non-tolerized post-translational modifications (PTMs) of self-protein). In the case of GFAP, the formation of GFAP-BDP by proteases such as calpain upon glial cell injury or death (by necrosis or apoptosis) following TBI fulfills this second requirement.

There are some limitations for our current study. Fro example, measurement of autoantibodies by western blot-based methods might have limited clinical applications [63]–[65]. In the future, it would also be valuable to develop an assay to measure anti-GFAP autoantibody levels in human blood reliably and quantitatively (e.g., an ELISA). In addition to measuring the presence of an autoantibody at a single time point, it would also be useful to quantify changes of anti-GFAP autoantibody over time after injury, as was done in this study (**Table 1**). In addition, anti-GFAP or GFAP-BDP autoantibodies are not limited to TBI, Thus caution should be exercised that anti-GFAP might not be a perfect subacute diagnostic of TBI as other factors can contribute to autoimmunity. Instead it might be a useful tool is showing the possibility that autoimmunity might play a role in individual and vulnerable subjects.

Okonkwo et al. [66] and Papa et al. [67] both recently described the GFAP/BDP antigens are detected in serum after mild-moderate TBI. These studies found GFAP-BDP antigen detectable in blood (serum, plasma), which is different from the present study where anti-GFAP autoAb is identified in subacute period post-TBI. In fact, these complementary studies show that significant GFAP-BDP levels are also released into circulation after moderate-mild TBI. For future studies, it will be important also to examine if autoantibody to GFAP is induced after mild to moderate TBI. While levels of released GFAP decline rapidly after TBI [48],[68], IgG-based autoantibodies could potentially last for days to months. Because of the long lasting nature of IgG, a test to detect anti-GFAP autoantibodies is likely to prolong the temporal window for assessing brain damage. Measuring patient titer over days to months following injury could provide additional information about patient outcome, progress or response to therapy.

The mechanistic effect of anti-GFAP autoantibodies is unclear at this point. Such autoantibodies could be beneficial, pathogenic, or of no consequence. The existence anti-GFAP autoantibodies in ∼16% of apparently normal patients suggests a benign role, at least as long as the BBB is intact. The negative correlation between fold change in TBI autoantibody levels and GOS-E at discharge suggests that increased anti-GFAP autoantibody levels may indicate more severe injury. This provocative finding should be verified by additional studies. Regardless of their biological role, this study has demonstrated that anti-GFAP autoantibodies could potentially serve as a novel subacute and chronic biomarker for TBI in humans.

## Supporting Information

Figure S1 **Purified GFAP protein blocked binding of TBI autoantibodies to the 50-38 kDa autoantigen.** Anti-GFAP antibody (Abcam) or serum from each of 3 TBI patients (Day 10) were pre-incubated in the absence (−), or presence of either 10 μg of purified GFAP or Tau protein prior to probing the human brain lysate blots.
(TIF)

Table S1 **Demographic and clinical data.** Details regarding the demographic and clinical data for severe TBI cases and normal controls included in this study are displayed (related to Figure 9).
(TIF)
