# Correction: Human Traumatic Brain Injury Induces Autoantibody Response against Glial Fibrillary Acidic Protein and Its Breakdown Products

**DOI:** 10.1371/journal.pone.0101712

**Published:** 2014-06-25

**Authors:** The PLOS ONE Staff

There is an error in the author affiliations. The first author Zhang-Z., fifth author Yang-Z and last author Wang-KK have incorrect affiliations. The correct affiliation for these three authors is #1 Department of Psychiatry, Center for Neuroproteomics and Biomarker Research, University of Florida, Gainesville, Florida, United States of America.

The second author J. Susie Zoltewicz, fourth author Kimberly J. Newsom, sixth author Boxuan Yang , eighth author Joy Guingab , ninth author Olena Glushakova also have incorrect affiliations. The correct affiliation for these three authors is #2 Banyan Biomarkers Inc., Alachua, Florida, United States of America.
