# Supplementary figures and images for: Human Traumatic Brain Injury Induces Autoantibody Response against Glial Fibrillary Acidic Protein and Its Breakdown Products
Source: PLoS One. 2014 Mar 25;9(3):e92698. doi: 10.1371/journal.pone.0092698 (PMC3965455; doi:10.1371/journal.pone.0092698)

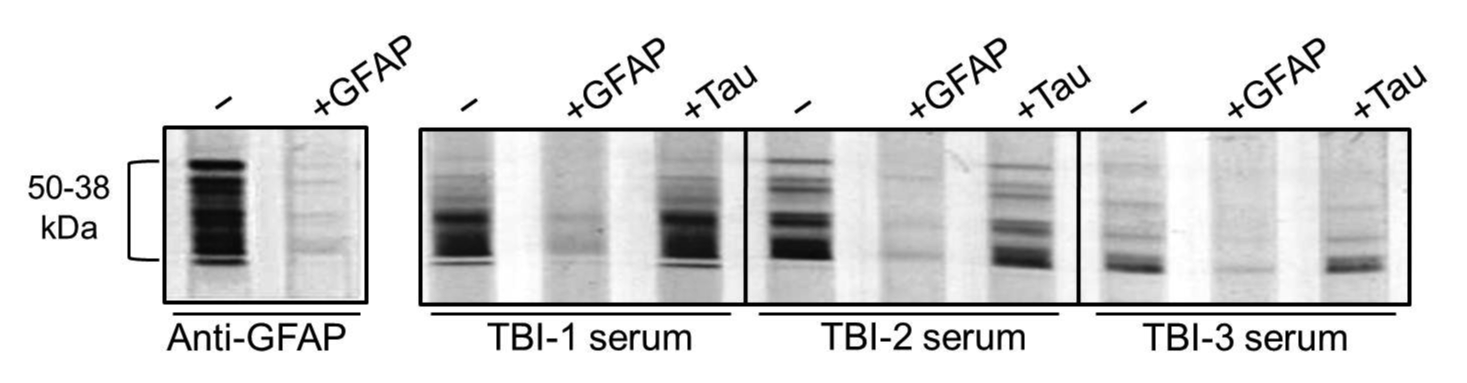

Supplement: Figure S1 — Purified GFAP protein blocked binding of TBI autoantibodies to the 50-38 kDa autoantigen. Anti-GFAP antibody (Abcam) or serum from each of 3 TBI patients (Day 10) were pre-incubated in the absence (−), or presence of either 10 μg of purified GFAP or Tau protein prior to probing the human brain lysate blots. (TIF) [file pone.0092698.s001.tif]

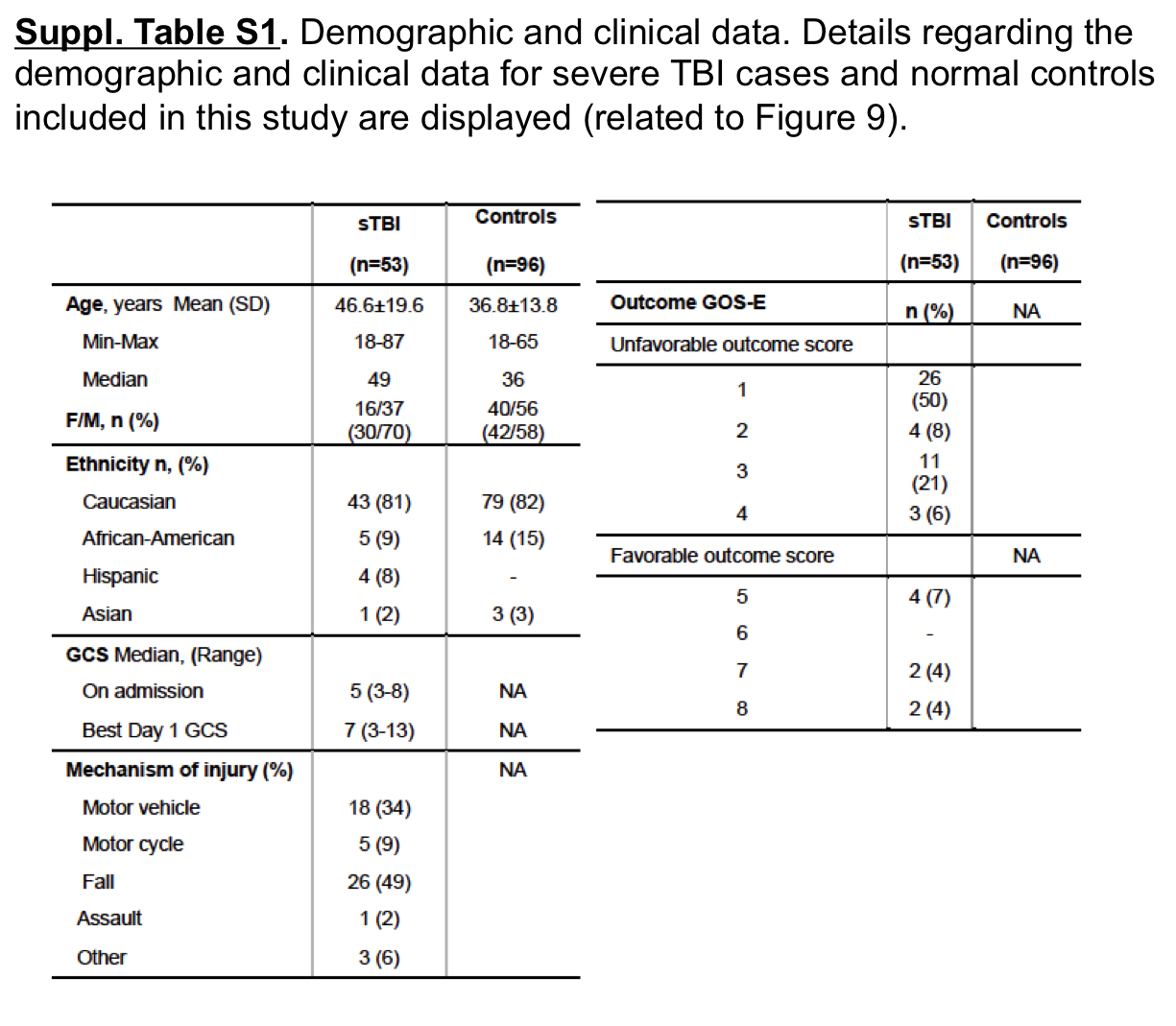

Supplement: Table S1 — Demographic and clinical data. Details regarding the demographic and clinical data for severe TBI cases and normal controls included in this study are displayed (related to Figure 9). (TIF) [file pone.0092698.s002.tif]
